# Human echinococcosis incidence in Canada: A retrospective descriptive study using administrative hospital and ambulatory visit data, 2000–2020

**DOI:** 10.14745/ccdr.v50i09a03

**Published:** 2024-09-05

**Authors:** Ayisha Khalid, Pia K Muchaal, Danielle A Julien

**Affiliations:** ^1^ Public Health Agency of Canada, Ottawa, ON; ^2^ Dalla Lana School of Public Health, University of Toronto, Toronto, ON

**Keywords:** echinococcosis, incidence, administrative data, Canada

## Abstract

**Background:**

Echinococcosis is a zoonotic disease caused by the ingestion of tapeworm eggs shed by canids. The potential recent establishment of a more virulent European-type strain may be impacting human echinococcosis in Canada, yet information is limited.

**Objective:**

Administrative hospital and ambulatory visit data were used to provide a baseline of human echinococcosis cases in Canada between 2000–2020.

**Methods:**

Canadian Institute of Health Information's Discharge Abstract Database, Hospital Morbidity Database and National Ambulatory Care Reporting System were combined to identify cases. Risk ratios (RR) by demographic factors and cumulative incidences (CIN) over place and time were calculated.

**Results:**

A total of 806 echinococcosis cases were identified in Canada between 2000–2020, for a mean annual CIN of 1.3 cases per million population. Over the two decades, the mean annual CIN of cases increased nationally (1.3–1.4 cases per million), in the Northwest Territories (6.3–9.1 cases per million), in Alberta (1.5–2.4 cases per million) and in the Atlantic provinces (0.2–0.6 cases per million). Those from the Territories had the highest risk of echinococcosis (RR 17.1; 95% confidence interval: 8.7–33.7).

**Conclusion:**

Though explanations are multifactorial, the new European-type strain may have a role in the small absolute increase in echinococcosis CIN in Canada observed over the study period. The CIN is likely underestimated and the validity of administrative data for analyzing zoonoses warrants investigation. Though this study contributes important awareness and a baseline, improved data are needed to clarify the effects of the new strain and inform public health response.

## Introduction

Echinococcosis is a rare zoonotic disease caused by infection with larval *Echinococcus* tapeworms ((1)). Tapeworm eggs are excreted in the feces of infected canids and can be ingested by humans through contaminated food, water or soil, or from close contact with infected animals ((1)). Compared to the general population, those who have frequent contact with canids, such as dog owners, can face increased risk of echinococcosis ((2)). Some Indigenous Peoples in Canada, Alaska, Russia and Siberia north of the Arctic Circle who practise traditional cultural activities, such as using sled dogs, hunting, fishing and gathering, may also face increased risk ((3–5)). In isolated areas, use of untreated surface water as a potable water source and inaccessible medical services can compound risk and contribute to more severe health outcomes ((5)).

Echinococcosis in humans occurs in two major forms. Cystic echinococcosis (CE), caused by *Echinococcus granulosus,* leads to hydatid cysts in organs, often the liver and lungs, that can impair physiological function ((1)). Alveolar echinococcosis (AE), caused by *Echinococcus multilocularis*, produces a tumour-like polycystic mass in organs, most often in the liver, that can infiltrate adjacent organs and tissues to produce distant metastases ((1)). Treatment generally requires surgical removal or chemotherapy ((1)). Echinococcosis is frequently under or misdiagnosed because the disease is rare, awareness is limited, both AE and CE have long incubation periods ranging 5–15 years and up to 60% of cases are asymptomatic ((1)).

While both AE and CE have been reported in Canada, AE was historically limited to the North American *E. multilocularis* strain and found almost exclusively in wildlife ((6)). In 2009, a new *E. multilocularis* strain more closely related to European strains was detected in a dog from British Columbia with no travel history outside of the province ((7)). Local canid transmission was identified thereafter in British Columbia as well as Alberta, Manitoba and Ontario ((8–11)). The first human case of AE with the European-type *E. multilocularis* strain was confirmed in Alberta in 2013 ((12)). Of six subsequent human cases in Alberta, molecular typing was available for five, all indicating the presence of the European-type strain ((13)).

European *E. multilocularis* strains have greater virulence and zoonotic potential than North American strains ((8)). Due to the potential establishment of the European-type strain in animal hosts, climate change, urbanization and anthropogenic activities, human AE is considered an emerging disease threat in Canada ((4,8)). Yet, knowledge about human echinococcosis in the country is limited. While AE is a provincially notifiable disease in Alberta, Ontario, Nunavut and the Northwest Territories, it is currently not nationally notifiable ((14)).

Absence of information on echinococcosis among people in Canada, exacerbated by limited awareness and underdiagnosis, as well as increasing evidence of emergence due to the detection of a more virulent strain, necessitates the use of alternative nationwide data sources to describe echinococcosis. This study leveraged administrative hospital and ambulatory visit data to provide a baseline for human echinococcosis in Canada between 2000–2020, relevant for increasing awareness and informing public health guidelines. Risk ratios (RRs) by demographic factors and incidences over place and time of echinococcosis cases were estimated. The authors hypothesized a higher incidence in 2011–2020 than 2000–2010, especially in isolated northern areas, due to the European-type *E. multilocularis* strain detected in 2009.

## Methods

### Data sources

To identify echinococcosis cases, three Canadian Institute for Health Information (CIHI) databases were combined: the Discharge Abstract Database (DAD), Hospital Morbidity Database (HMDB) and National Ambulatory Care Reporting System (NACRS). These databases collect data on an annual basis corresponding to the fiscal year (April 1 of one year to March 31 of the following year) ((15)). The DAD and HMDB databases similarly capture national administrative, clinical and basic demographic information on hospital inpatient events, however, the DAD does not include data from Québec ((15)). The NACRS contains complete or partial data on hospital-based and community-based ambulatory care from Alberta, British Columbia, Manitoba, Nova Scotia, Ontario, Prince Edward Island, Québec, Saskatchewan and Yukon ((15)).

### Eligibility criteria

Diagnoses in CIHI databases use the ninth or tenth revision of the World Health Organization's International Classification of Diseases (ICD-9 and ICD-10) ((16,17)). Cases were defined as patients visiting hospital or ambulatory care for whom the main responsible diagnosis or one of the first five discharge diagnoses was echinococcosis (ICD-9 codes 122.0 to 122.9; ICD-10 codes B67.0 to B67.9).

To derive the echinococcosis cases dataset, the available DAD, HMDB and NACRS data were first merged. Records of cases in years with incomplete data due to collection on a fiscal year basis were removed. Then, duplicates and records describing readmissions for echinococcosis for the same case were removed to align with the goal of estimating incidence. Specifically, the first chronological record was retained, regardless of which database it came from and subsequent records were excluded. The CIHI databases contain encrypted health card numbers that were used to find records for the same case. The SAS Enterprise Guide® 7.1 software for Microsoft Windows was used to merge data.

### Data analysis

Descriptive analyses were used to characterize echinococcosis cases by infecting *Echinococcus* species, sex, age group and region and province/territory (P/T) of health card issuance. Bivariate analyses were used to determine the RR, with 95% confidence intervals (CI), of echinococcosis by sex, age group, region and P/T. Québec was excluded from RR calculations to avoid skewed comparisons, as data from the province were only available from the HMDB for the first half of the study period (2000–2010). Population estimates from Statistics Canada's 2011 Census of Population were used as denominators to compute RRs ((18)).

The cumulative incidence (CIN) of echinococcosis cases over 2000–2020 at the national, regional and P/T levels was calculated using annual population estimates (fourth quarter) from Statistics Canada as denominators ((19)). The mean annual CIN was calculated by taking an average of the yearly CIN of echinococcosis cases. Québec was excluded from CIN calculations. Data were analyzed using R Statistical Software (v4.1.1; R Core Team 2021) and QGIS Geographic Information System 3.8 was used to map CIN.

## Results

### Characteristics and risk ratios

**Figure 1:**
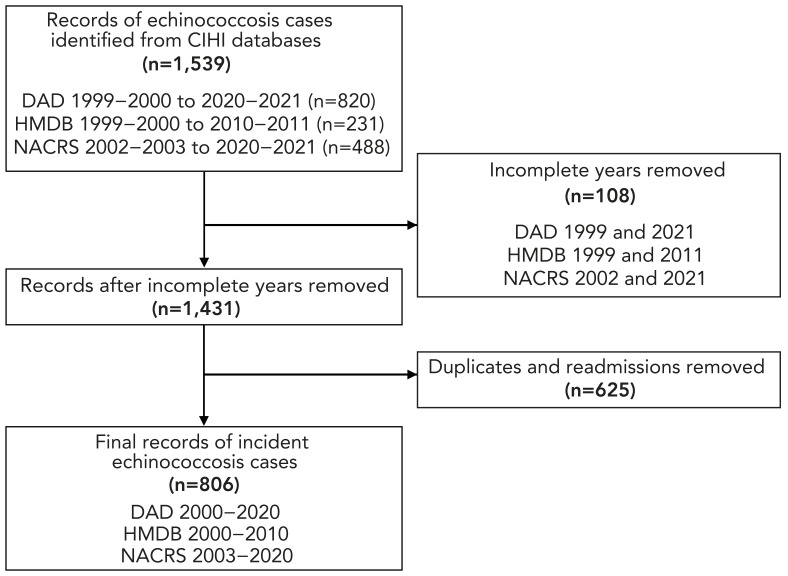
Flow diagram of incident human echinococcosis hospital and ambulatory care visits, as cases, record selection, Canadian Institute for Health Information, 2000–2020 Abbreviations: CIHI, Canadian Institute for Health Information; DAD, Discharge Abstract Database; HMDB, Hospital Morbidity Database; NACRS, National Ambulatory Care Reporting System

The final dataset comprised 806 records of incident echinococcosis cases in Canada between 2000–2020 (**Figure 1**). The demographic characteristics of cases and RRs are presented in **Table 1**. Of the 806 cases, most were unspecified (n=669; 82.3%), followed by *E. granulosus* (n=111; 13.7%) and *E. multilocularis* (n=33; 4.1%). The largest proportion of cases (n=371; 46.0%) were from Ontario. Females comprised over half of cases (n=501; 62.2%) and were at 1.6 (95% CI: 1.4–1.8) times higher risk of echinococcosis compared to males. While most cases were aged 35–54 years (n=265; 32.9%), those over 75 years of age had the highest risk, at 5.6 (95% CI: 3.9–8.0) times higher than those aged 0–14 years.

**Table 1:** Characteristics and risk ratios of echinococcosis hospital and ambulatory care visits, as cases, in Canada, Canadian Institute for Health Information^a^, 2000–2020 (n=806)

Characteristics	Echinococcosis cases	
	n (%)	Risk ratios (95% CI)
***Echinococcus* species^b^**		
*E. multilocularis*	33 (4.1)	N/A
*E. granulosus*	111 (13.7)	N/A
Unspecified	669 (82.3)	N/A
**Sex**		
Male	305 (37.8)	1.0
Female	501 (62.2)	1.6 (1.4–1.8)
**Age group**		
0–14 years	42 (5.2)	1.0
15–34 years	193 (23.9)	3.0 (2.1–4.1)
35–54 years	265 (32.9)	3.6 (2.6–5.0)
55–74 years	211 (26.2)	4.0 (2.9–5.6)
≥75 years	95 (11.8)	5.6 (3.9–8.0)
**Geography**		
**Atlantic region**	**19 (2.4)**	**1.0**
Prince Edward Island	1 (0.1)	1.0
New Brunswick	7 (0.9)	1.3 (0.2–10.3)
Newfoundland and Labrador	5 (0.6)	1.3 (0.2–11.3)
Nova Scotia	6 (0.7)	0.9 (0.1–7.4)
**Eastern region**	**436 (54.1)**	**3.5 (2.2–5.6)^c^**
Ontario	371 (46.0)	3.9 (0.6–27.9)
Québec	65 (8.1)	N/A^c^
**Western region**	**336 (41.7)**	**4.0 (2.5–6.4)**
Alberta	155 (19.2)	5.8 (0.8–41.3)
British Columbia	102 (12.7)	3.2 (0.4–22.6)
Manitoba	37 (4.6)	4.2 (0.6–30.3)
Saskatchewan	42 (5.2)	5.5 (0.8–40.1)
**Territories region**	**15 (1.9)**	**17.1 (8.7–33.7)**
Northwest Territories	7 (0.9)	22.9 (2.8–186.4)
Nunavut	4 (0.5)	17.0 (1.9–152.4)
Yukon	4 (0.5)	16.0 (1.8–143.4)

Abbreviations: CI, confidence interval; *E., Echinococcus*; N/A, not applicable

^a^ Including the Discharge Abstract Database (2000–2020), Hospital Morbidity Database (2000–2010) and National Ambulatory Care Reporting System (2003–2020)

^b^ Seven cases reported with multiple diagnoses of echinococcosis in the same record. Therefore, *Echinococcus* species sums to 813 cases

^c^ Québec excluded, as data for the province were unavailable between 2011–2020

Cases with a health card issued from the Territories region (Northwest Territories, Nunavut and Yukon) had a much higher risk of echinococcosis (RR 17.1; 95% CI: 8.7–33.7) compared to the Atlantic region (New Brunswick, Newfoundland and Labrador, Nova Scotia and Prince Edward Island). Cases from the Northwest Territories had the highest risk of echinococcosis in the country, at 22.9 (95% CI: 2.8–186.4) times that of Prince Edward Island. Among provinces, cases from the Western region (Alberta, British Columbia, Manitoba and Saskatchewan) (RR 4.0; 95% CI: 2.5–6.4), compared to the Atlantic region, had the highest risk of echinococcosis.

### Cumulative incidence

As shown in **Table 2**, the mean annual CIN of echinococcosis cases in Canada between 2000–2020 was 1.3 cases per million population. There was a slight absolute increase over the two decades nationally, from 1.3 cases per million between 2000–2010 to 1.4 cases per million between 2011–2020. The mean annual CIN of cases diagnosed as *E. multilocularis* increased very slightly over the two decades (0.05–0.06 cases per million), while cases diagnosed as *E. granulosus* decreased very slightly (0.19–0.18 cases per million). Detailed count and CIN by *Echinococcus* species, geography and year is provided in the **Appendix** as Supplemental material.

**Table 2:** Mean annual cumulative incidence, per million population, of echinococcosis hospital and ambulatory care visits, as cases, over place and time in Canada, Canadian Institute for Health Information^a^, 2000–2020 (n=741)

Geography	Mean annual CIN (per million population)		
	Overall (2000–2020)	First decade (2000–2010)	Second decade (2011–2020)
**National**	**1.3**	**1.3**	**1.4**
*E. multilocularis*	0.06	0.05	0.06
*E. granulosus*	0.20	0.19	0.18
Unspecified	1.1	1.1	1.1
**Atlantic region**	**0.4**	**0.2**	**0.6**
New Brunswick	0.4	0.4	0.5
Newfoundland and Labrador	0.5	0.4	0.6
Nova Scotia	0.3	0.1	0.5
Prince Edward Island	0.3	0	0.6
**Eastern region**	**1.3^b^**	**1.4^b^**	**1.3^b^**
Ontario	1.3	1.4	1.3
Québec	N/A^b^	N/A^b^	N/A^b^
**Western region**	**1.6**	**1.6**	**1.6**
Manitoba	1.4	1.4	1.5
Saskatchewan	1.9	2.0	1.8
Alberta	1.9	1.5	2.4
British Columbia	1.1	1.4	0.8
**Territories region**	**6.2**	**6.8**	**5.6**
Northwest Territories	7.6	6.3	9.1
Nunavut	5.8	8.6	2.6
Yukon	5.2	5.3	5.1

Abbreviations: CIN, cumulative incidence; *E., Echinococcus*; N/A, not applicable

^a^ Including the Discharge Abstract Database (2000–2020), Hospital Morbidity Database (2000–2010) and National Ambulatory Care Reporting System (2003–2020)

^b^ Québec excluded as data for the province were unavailable between 2011–2020

**Figure 2:**
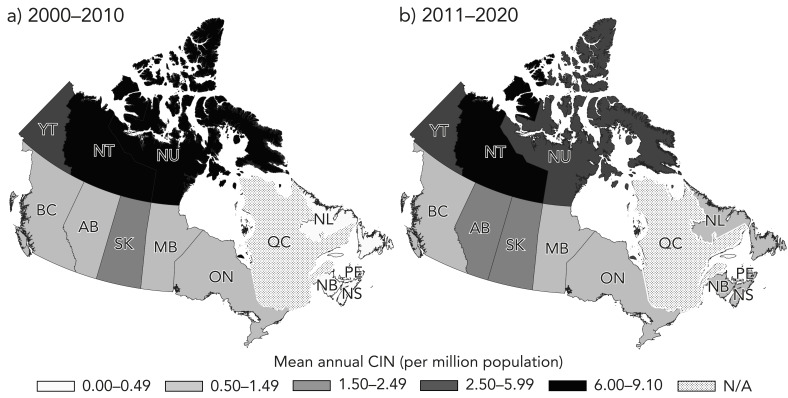
Map of the mean annual cumulative incidence, per million population, of echinococcosis hospital and ambulatory care visits, as cases, by province and territory in Canada between 2000–2010 (n=364) and 2011–2020 (n=377), Canadian Institute for Health Information^a^ Abbreviations: CIN, cumulative incidence; N/A, not applicable because Québec was excluded due to unavailable data between 2011–2020 ^a^ Including the Discharge Abstract Database (2000–2020), Hospital Morbidity Database (2000–2010) and National Ambulatory Care Reporting System (2003–2020)

The Territories region had the absolute highest mean annual CIN of echinococcosis cases overall, at 6.2 cases per million (Table 2). Though case counts were low, over the two decades, there was an increase in the Northwest Territories (6.3–9.1 cases per million) but decreases in Nunavut (8.6–2.6 cases per million) and Yukon (5.3–5.1 cases per million) (**Figure 2**), resulting in a regional absolute decrease in mean annual CIN from 6.8 to 5.6 cases per million.

Among provinces, the Western region had the absolute highest mean annual CIN of echinococcosis cases, at 1.6 cases per million (Table 2). In Alberta, the mean annual CIN increased over the two decades from 1.5 to 2.4 cases per million (Figure 2). The Eastern region, which only included Ontario due to limited data from Québec, had the second absolute highest mean annual CIN of echinococcosis cases overall, at 1.3 cases per million. In the Atlantic region, the mean annual CIN was low overall at 0.4 cases per million. However, though case counts were low, each province in the Atlantic region experienced an increase in the mean annual CIN of cases over the two decades, resulting in a regional absolute increase from 0.2 to 0.6 cases per million.

## Discussion

This study used administrative data to describe echinococcosis incidence and risk in Canada between 2000–2020. The mean annual CIN of echinococcosis in Canada over the study period was rare at 1.3 cases per million, which was slightly lower than the 1.5 cases per million reported by the European Surveillance System in 2020 ((20)). Between 2001–2005 in Canada, Gilbert *et al.* ((21)) found a lower mean incidence than this study, at 0.72 echinococcosis hospitalizations per million. Schurer *et al.* ((22)) found a median annual incidence between 2002–2011 of 1.4 echinococcosis hospital and ambulatory visits per million. The Gilbert *et al*. ((21)) estimate may be lower because they only used HMDB and restricted to cases with only a first or second discharge diagnosis of echinococcosis. Schurer *et al.* ((22)) used the DAD and NACRS and included cases with an echinococcosis diagnosis in any of the 25 available discharge diagnoses. This study's use of the DAD, HMDB and NACRS may have been beneficial for capturing hospital and ambulatory visits more completely.

The results indicated an absolute increase, though small, in the mean annual CIN of echinococcosis cases in Canada between 2011–2020 compared to 2000–2010. Whether this was due to the European-type *E. multilocularis* strain first detected in Canada in 2009 remains unclear, as the species-level diagnosis for most cases was unspecified. Distinguishing *E. multilocularis* from *E. granulosus* in humans is not only an epidemiological but also a clinical necessity, as there are differences in prognosis, treatment, intermediate hosts and regional prevalence ((1)). Species-level diagnosis is complex, involving imaging, microscopy and serology ((1)). Serology is required for early stages of infection, while later stages may be diagnosed through histopathology ((23)). For people in Canada, confirmatory diagnosis of *E. multilocularis* demonstrating larval tapeworms in histopathology samples can require species-specific polymerase chain reaction (PCR) or serologic testing in some provinces ((24)). While this PCR is done at a limited number of laboratories across North America, approved serologic testing is only performed at the Institute of Parasitology in Switzerland ((23)). Studies have recommended that accessible and standardized testing optimized for circulating species of *Echinococcus* and increased awareness of clinical signs among physicians and veterinarians in endemic regions, would help improve prognosis and surveillance in Canada ((12,22)).

Over the two decades, there was a notable absolute increase in mean annual CIN of echinococcosis cases in the Northwest Territories. Having a health card from any of the three Territories also posed the highest risk of echinococcosis. The overall mean annual CIN for the Territories region (6.2 cases per million) was closer to that which has been recorded in European countries considered endemic for echinococcosis, like Luxembourg (4.8 cases per million) ((20,25)). Northern parts of Canada may be at higher risk of echinococcosis due to some populations hunting, consuming untreated surface water, keeping dogs as pets and working animals and harvesting potentially contaminated food ((5,21,22)).

There was also an absolute increase in the mean annual CIN of echinococcosis cases in Alberta over the two decades. Alberta had the second-highest number of cases diagnosed as *E. multilocularis* following Ontario despite having a substantially smaller population. Between 2013–2020, 17 cases of human AE were identified in Alberta, all likely locally acquired and all five of the cases with molecular typing results showing presence of the European-type strain ((13)). Among coyotes in urban areas of Alberta, studies have highlighted an increasing prevalence of *E. multilocularis,* ranging from 25% between 2009–2011 ((26)) to 65% between 2016–2018 ((8)), with histology results from the region often confirming the presence of the European-type strain ((9,27)).

The Atlantic region had the lowest mean annual CIN of echinococcosis cases, but it increased slightly over the two decades. In the past 30 to 40 years, coyotes have reportedly expanded their range from the Great Lakes region of southern Canada into eastern Canada ((28)). A recent study recorded the first ever instances of *E. canadensis,* a subtype of *E. granulosus,* in free-ranging wildlife in Atlantic Canada (one coyote and four moose), suggesting that coyote natural range expansion has a role in enabling the lifecycle of *Echinococcus* tapeworms in the region ((28)).

Similar to previous Canadian literature, females in this study had a significantly higher risk of echinococcosis compared to males, warranting further investigation ((21,22)). Older age was also associated with a significantly higher risk of echinococcosis; however, this may be due to the long incubation period preceding clinical manifestations of the disease ((1)).

In the absence of national reporting and surveillance of echinococcosis in Canada, CIHI databases were explored as an option for monitoring cases of this potentially increasing zoonosis. Administrative data are useful for investigating disease epidemiology, as they are population-based, timely, accessible, provide large sample sizes and have broad jurisdictional coverage. However, administrative data are not collected for research purposes and may have quality and reliability issues ((29)). There is value in prioritizing future studies to examine the validity of administrative data sources for studying zoonoses in the future.

## Limitations

There are some limitations of this study. Though it was the only available nationwide data source for echinococcosis, using hospital and ambulatory data to estimate incidence likely resulted in an underestimation. Echinococcosis is rare with a long incubation period, increasing the chance of under or misdiagnosis and most, but not all, symptomatic infections require medical attention ((1)). The incidence is also likely underestimated, both overall and for the Eastern region especially, because data for Québec were unavailable between 2011–2020 and Québec contributed 15% of all cases between 2000–2010.

Administrative data can have quality and reliability concerns and often lack information on potentially relevant indicators. For example, we did not have data on travel history and as echinococcosis has a long incubation period, this may have been relevant for understanding local disease acquisition. Additionally, not all P/Ts have mandated reporting to NACRS; those with mandated reporting may have contributed more echinococcosis cases than those without.

The RRs of echinococcosis for the Territories had wide CIs, likely because of small population sizes and indicate imprecision. Due to the small population sizes, the CIN for the Territories were also unstable.

## Conclusion

This study fills an important gap by contributing a baseline for human echinococcosis in Canada between 2000–2020. Although echinococcosis is rare, there was a small absolute increase in the mean annual CIN of cases nationally between 2011–2020 compared to 2000–2010. Further research is needed to determine the role of the new European-type *E. multilocularis* strain, in addition to climate change, urbanization and anthropogenic activity, on disease burden. Improved and complete data are needed to understand differences across provinces and territories, in order to inform engagement with and guidelines for, public health partners, key risk groups and the general public. Research investigating the validity of administrative data for zoonoses is also warranted.

## Appendix

Supplemental material is available upon request to the author: ayisha.khalid@mail.utoronto.ca

Aggregated case count and incidence by *Echinococcus* species, geography and year
